# Supplementary figures and images for: A portable, low-cost device for precise control of specimen temperature under stereomicroscopes
Source: PLoS One. 2020 Mar 11;15(3):e0230241. doi: 10.1371/journal.pone.0230241 (PMC7065815; doi:10.1371/journal.pone.0230241)

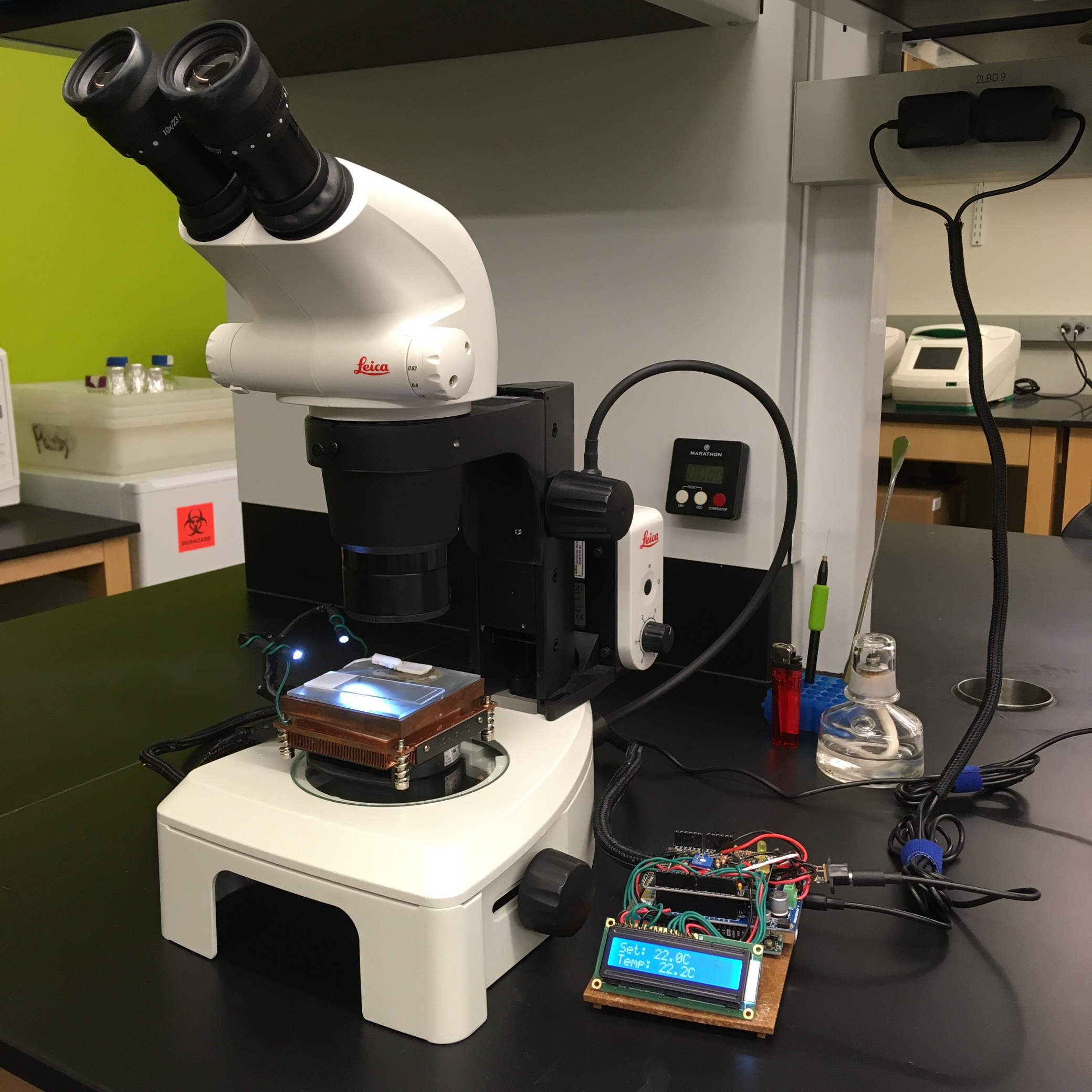

Supplement: S1 Fig — (JPEG) [file pone.0230241.s001.jpeg]

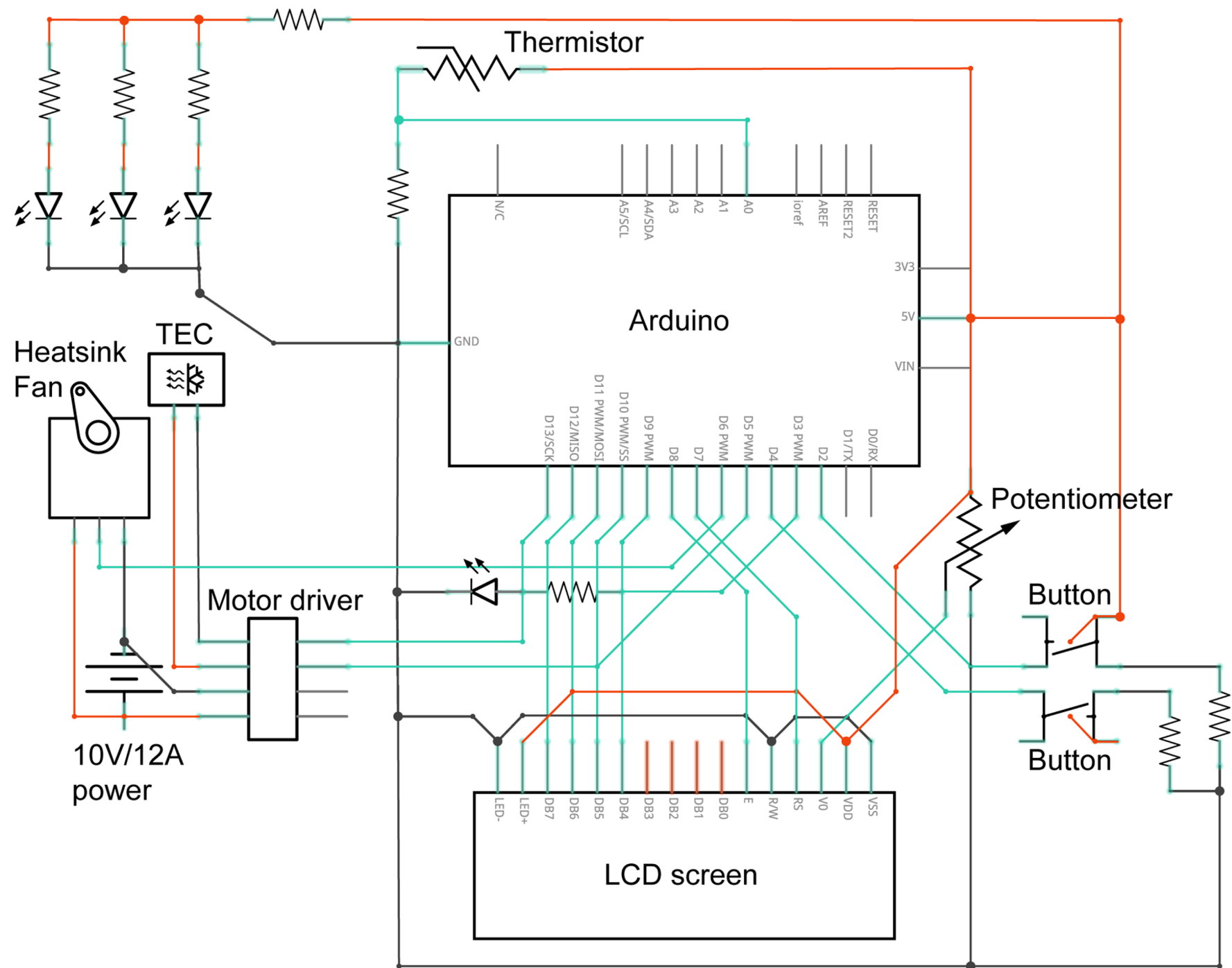

Supplement: S2 Fig — Orange lines indicate positive wires, black indicate ground, and teal indicate Arduino input/output. (PDF) [file pone.0230241.s002.pdf]
