# Supplementary material for: A portable, low-cost device for precise control of specimen temperature under stereomicroscopes
Source: PLoS One. 2020 Mar 11;15(3):e0230241. doi: 10.1371/journal.pone.0230241 (PMC7065815; doi:10.1371/journal.pone.0230241)
Supplement: S1 File — (PDF) [file pone.0230241.s003.pdf]

Partslist

Part	Cat. / Part No.	Quant.	Source	Cost
TEC (Peltier)	64975-502	1	Laird Technologies	$67.63
thermistor	SA2F-TH-44031-40	1	Omega	$60.00
Arduino starter kit	Arduino Starter Kit - ENG	1	Arduino.com	$99.90
	Arduino Uno Rev. 3	1		
	Alphanumeric LCD, 16x2 characters	1		
	Breadboard, 400 points	1		
	Solid core jumper wires, 22 gauge	70		
	Wooden base	1		
	220 Ohm resistor	3		
	Pushbuttons	2		
	Potentiometer, 10k Ohms	1		
	LED, bright white	1		
motor driver	SHIELD-MD10	1	Cytron	$13.78
protoshield	PROTO-01	1	OSEPP	$6.99
bottom heatsink	Heatsink R18	1	Dynatron	$29.59
top heatsink	-	1	custom made	-
thermal paste	XMS99	2	Arctic Silver 5	11.99
Arduino power supply	101-60-182	1	SainSmart	$7.99
mirror (square 3x3”)	ASIN B004HGODQU	1	Amazon.com	$5.58
etching cream	15-0200	1	Armour Products	$21.09
TEC power supply (12V/10A output)	ASIN B00Z9X4GLW	1	Amazon.com	$17.99
power supply cable (5.5mm x 2.1mm)	ASIN B072BXB2Y8	1	Amazon.com	$6.98
				$349.51
